# Evaluating normalized registration and preprocessing methodologies for the analysis of brain MRI in pediatric patients with shunt-treated hydrocephalus

**DOI:** 10.3389/fnins.2024.1405363

**Published:** 2024-05-30

**Authors:** Renee-Marie Ragguett, Roy Eagleson, Sandrine de Ribaupierre

**Affiliations:** ^1^School of Biomedical Engineering, Western University, London, ON, Canada; ^2^Department of Electrical and Computer Engineering, Western University, London, ON, Canada; ^3^Centre for Brain and Mind, Western University, London, ON, Canada; ^4^Department of Clinical Neurological Sciences, Schulich School of Medicine, Western University, London, ON, Canada

**Keywords:** registration, normalization, pediatric, hydrocephalus, non-linear registration, MRI

## Abstract

**Introduction:**

Registration to a standardized template (i.e. “normalization”) is a critical step when performing neuroimaging studies. We present a comparative study involving the evaluation of general-purpose registration algorithms for pediatric patients with shunt treated hydrocephalus. Our sample dataset presents a number of intersecting challenges for registration, representing the potentially large deformations to both brain structures and overall brain shape, artifacts from shunts, and morphological differences corresponding to age. The current study assesses the normalization accuracy of shunt-treated hydrocephalus patients using freely available neuroimaging registration tools.

**Methods:**

Anatomical neuroimages from eight pediatric patients with shunt-treated hydrocephalus were normalized. Four non-linear registration algorithms were assessed in addition to the preprocessing steps of skull-stripping and bias-correction. Registration accuracy was assessed using the Dice Coefficient (DC) and Hausdorff Distance (HD) in subcortical and cortical regions.

**Results:**

A total of 592 registrations were performed. On average, normalizations performed using the brain extracted and bias-corrected images had a higher DC and lower HD compared to full head/ non-biased corrected images. The most accurate registration was achieved using SyN by ANTs with skull-stripped and bias corrected images. Without preprocessing, the DARTEL Toolbox was able to produce normalized images with comparable accuracy. The use of a pediatric template as an intermediate registration did not improve normalization.

**Discussion:**

Using structural neuroimages from patients with shunt-treated pediatric hydrocephalus, it was demonstrated that there are tools which perform well after specified pre-processing steps were taken. Overall, these results provide insight to the performance of registration programs that can be used for normalization of brains with complex pathologies.

## 1 Introduction

Pediatric hydrocephalus is a disease characterized by a complex set of neurological indications—in particular a high volume of cerebrospinal fluid in the cerebral ventricles. While there is interest in studying pediatric hydrocephalus using neuroimaging techniques to learn more about the disease, working with these images may prove to be difficult given the potentially large pathology induced deformations and artifacts from surgical treatment (e.g., shunts) (Ou et al., 2014; Patel et al., 2017). When performing neuroimaging studies, a common goal is to be able to compare findings between participants. In order to accomplish this, the neuroimages must be registered to a standard stereotaxic space (i.e., spatial normalization) such as the Montreal Neurological Institute (MNI) space using a template image (e.g., MNI-152), such that there is a one-to-one correspondence between images (Mazziotta et al., 1995). Poor normalizations, wherein there is suboptimal alignment of brain regions relative to the template image, can have a variety of impacts on the results of neuroimaging studies. For example, in functional magnetic resonance imaging studies, poor normalizations can result in decreased sensitivity and false negatives wherein observed effects could be driven by structural rather than functional differences (Crinion et al., 2007). Therefore, it is not surprising that image registration is a non-trivial task, and there has been ongoing interest in assessing the accuracy of various programs used for registration (Crinion et al., 2007; Klein et al., 2009; Ou et al., 2014).

Image registration can be characterized by the possible transformation into two categories: linear and nonlinear. Linear registration in 3D can perform translations, rotations, scales, and skews in three directions (x, y, and z). In contrast, non-linear registration allows for deformations. Normalization can take advantage of a combination of both methods wherein there can be an initial linear registration followed by a non-linear registration. A number of freely available neuroimaging and medical imaging programs include functions for performing both these registrations (e.g., FMRIB Software Library [FSL^1^], and Statistical Parametric Mapping [SPM^2^]) (Smith et al., 2004; Ashburner, 2007).

Difficulty in performing registrations can occur for a variety of reasons. Ou et al. (2014), have operationalized the potential difficulties into four overarching challenges, which include: inter-participant anatomical variation, intensity and noise differences, protocol and field-of-view differences, and pathology induced missing correspondence. Often, there can be many of these challenges present in one dataset. For example, many of these challenges can be observed particularly in clinical pediatric populations wherein there can be pathology induced missing correspondence in addition to age-based anatomical variation (Courchesne et al., 2000).

There exist various methods to improve normalization accuracy with pathological brains. Tang et al. (2017) have characterized these methods into three overarching categories which include: masking, pathology simulation, and inpainting (Tang et al., 2017). Specifically, cost function masking, wherein a region of non-correspondence in the image is masked, has been shown to result in more accurate registrations (Brett et al., 2001). The generation of masks, however, can be incredibly time consuming, particularly in cases wherein the regions of interest cannot be accurately segmented automatically thus requiring manual segmentation, and there are many participants. Further, even when segmentation can be completed automatically, many segmentation methods are computationally intensive. Indeed, segmenting the enlarged ventricles such as those found in hydrocephalus can pose a challenge for many programs that perform automated segmentations. As such there has been an increase in exploration of solutions with increased accuracy in segmentation and concurrently decreased processing time (Shao et al., 2019; Quon et al., 2020). As a result, there is interest in general purpose normalization pipelines which can be utilized for these complex images that can produce accurate results without extensive manual work and computationally expensive processes. Furthermore, given the heterogeneity of the data due the large variation in ventricle size, having a single pipeline that can apply to all patients would be beneficial. This is particularly pertinent as the data associated with medical images are becoming increasingly large (Scholl et al., 2011).

To date, there have been no studies assessing the efficacy of various normalization pipelines for pediatric hydrocephalus. The normalization of neuroimages in those with shunt-treated pediatric hydrocephalus provides a unique series to study as these images represent a variety of challenges including non-correspondence and artifacts from shunt treatment, potentially large pathology induced deformities in the ventricles and surrounding tissues, and age-based anatomical variation. Indeed, once treated with a ventriculoperitoneal shunt, the ventricles can range from being smaller than normal, to staying extremely large depending on when the shunt is inserted in the life of the child, and what type of valve is used. The objective of the current study is to assess the accuracy of a variety of freely available registration programs after preprocessing steps in pediatric hydrocephalus, a population who has a wide variation in brain imaging, and explore the impact of ventriculomegaly.

## 2 Methods

### 2.1 Participants

**Figure 1:**
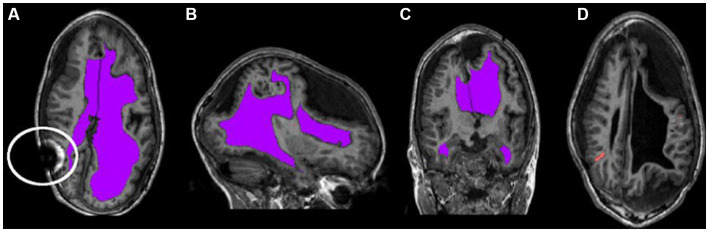
Characteristic asymmetries seen in pediatric hydrocephalus. **(A–C)** The ventricles segmented in purple and highlight the potentially non-typical brain shape. The circle in **(A)** outlines an artifact that can occur as a result of the shunt. **(D)** The catheter segmented in orange.

Clinically stable children with hydrocephalus treated by ventriculoperitoneal shunts were recruited from a pediatric neurosurgical outpatient clinic in London, Ontario, Canada. Written informed consent and assent was obtained from all parents and children, respectively. Approval was obtained from our institutional research ethics board. Inclusion criteria included patients with hydrocephalus within the first two years of life or intraventricular hemorrhage at birth. Patients were not eligible for the study if they had a programmable shunt or any other contraindications for MRI. Figure 1 shows characteristics of neuroimages of those with pediatric hydrocephalus that could impact normalization.

### 2.2 Magnetic resonance imaging acquisition

Neuroimages were acquired from a Siemens MAGNETOM Prisma 3-Tesla MRI scanner with a 32-channel head coil. A whole brain T1-weighted image was acquired using the three-dimensional magnetization-prepared rapid gradient-echo (MPRAGE) sequence (Repetition time [TR] = 2,300 ms; Echo time [TE] = 2.93 ms; Inversion time [TI] = 900 ms; Flip Angle = 9°; Matrix Size = 256 × 256, Number of Slices = 160; Field of View [FOV] = 256 mm; Resolution = 1.0 × 1.0 × 1.0 mm^3^).

### 2.3 Image preprocessing

**Figure 2:**
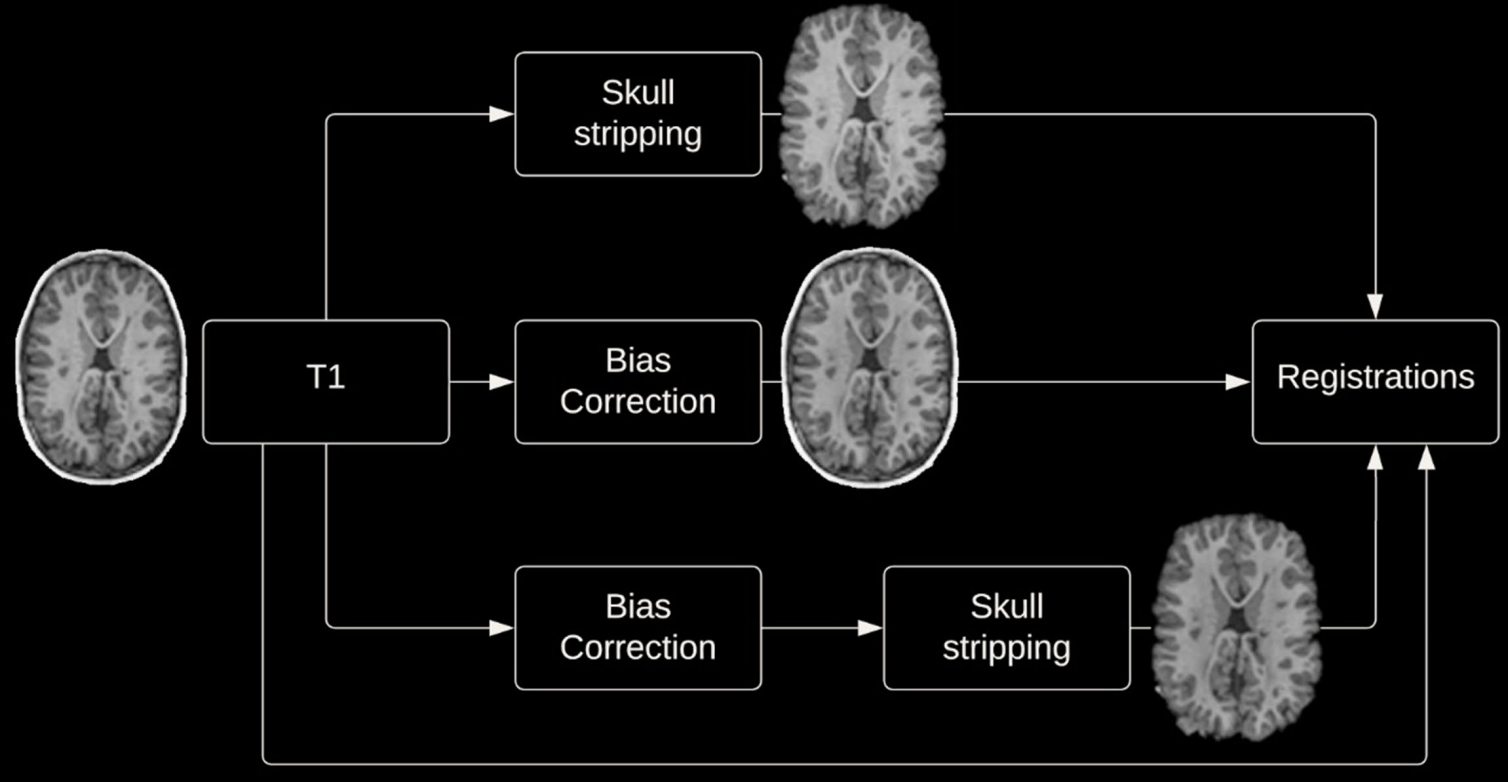
Four preprocessing options.

Given the potential impact of image preprocessing on registration accuracy, various preprocessing steps were performed. Registrations were performed with and without skull-stripping, and with or without bias correction (view Figure 2 for preprocessing pipeline). Registration using the DARTEL Toolbox was performed only with whole-brain data as segmentation of the tissue types is required to run DARTEL and SPM’s segmentation tool will remove the non-brain tissues.

#### 2.3.1 Skull stripping

Removal of non-brain tissues was completed using the Brain Extraction Tool (BET) from FMRIB Software Library (FSL) version 6.0 (see text footnote 1) (Smith, 2002). In order to achieve an accurate brain extraction given the large deformities present in the dataset, various BET parameters were tuned, and manual removal of non-brain structures was performed following BET on a per subject basis.

#### 2.3.2 Bias correction

Bias correction to correct for intensity inhomogeneities was performed using N4 bias field correction from Advanced Normalization Tools (ANTs)^3^ (Tustison et al., 2010).

### 2.4 Image registration

Images were both registered to the 1 × 1 × 1 mm^3^ MNI-152 nonlinear 6th generation template. Additionally, images were registered to the age-specific NIHPD symmetric pre- to mid-puberty (7.5 years to 13.5 years) 1 × 1 × 1 mm^3^ pediatric template, followed by registration to the aforementioned MNI-152 template. This additional registration was performed as it has been suggested that registering an age specific template could produce more accurate registrations (Wilke et al., 2002; Fonov et al., 2011). Image registration using the DARTEL Toolbox differed from the aforementioned process, firstly DARTEL creates a groupwise template image wherein each participant’s neuroimage is registered to the groupwise template, then these images can be normalized to MNI space.

#### 2.4.1 Registration program details

A variety of freely available programs commonly used for neuroimaging analysis were chosen. The selected programs employ a variety of registration algorithms and implementations. All registrations were implemented using default parameters (view Appendix 1), except for FLIRT wherein two iterations were used, one with the default parameters and a second with a reduced angular range for initial optimization (FLIRT 2 and FNIRT 2 represent linear and non-linear registrations completed with a reduced angular range during the linear registration step). View Table 1 for the programs used. Characteristics of the algorithms including deformation model, similarity, and regularization, have been summarized by Ou et al. (2014) and Klein et al. (2009).

**Table 1:** Registration programs assessed.

Function, *Program*
Deformable Registration via Attribute Matching and Mutual-Saliency Weighting (DRAMMS), *DRAMMS Deformable Image Registration Toolbox* (https://www.nitrc.org/projects/dramms) (Ou et al., 2011)
Diffeomorphic Anatomical Registration using Exponentiated Lie algebra (DARTEL), *Statistical Parametric Mapping (SPM)* (Ashburner, 2007)
FMRIB’s Linear Image Registration Tool (FLIRT), *FMRIB Software Library (FSL)* (Jenkinson et al., 2002)
FMRIB’s Non-Linear Image Registration Tool (FNIRT), *FMRIB Software Library (FSL)* (Smith et al., 2004)
Symmetric Image Normalization (SyN), *Advanced Normalization Tools (ANTs)* (http://stnava.github.io/ANTs/) (Avants et al., 2011)

### 2.5 Data analyses

#### 2.5.1 Region selection and verification

**Figure 3:**
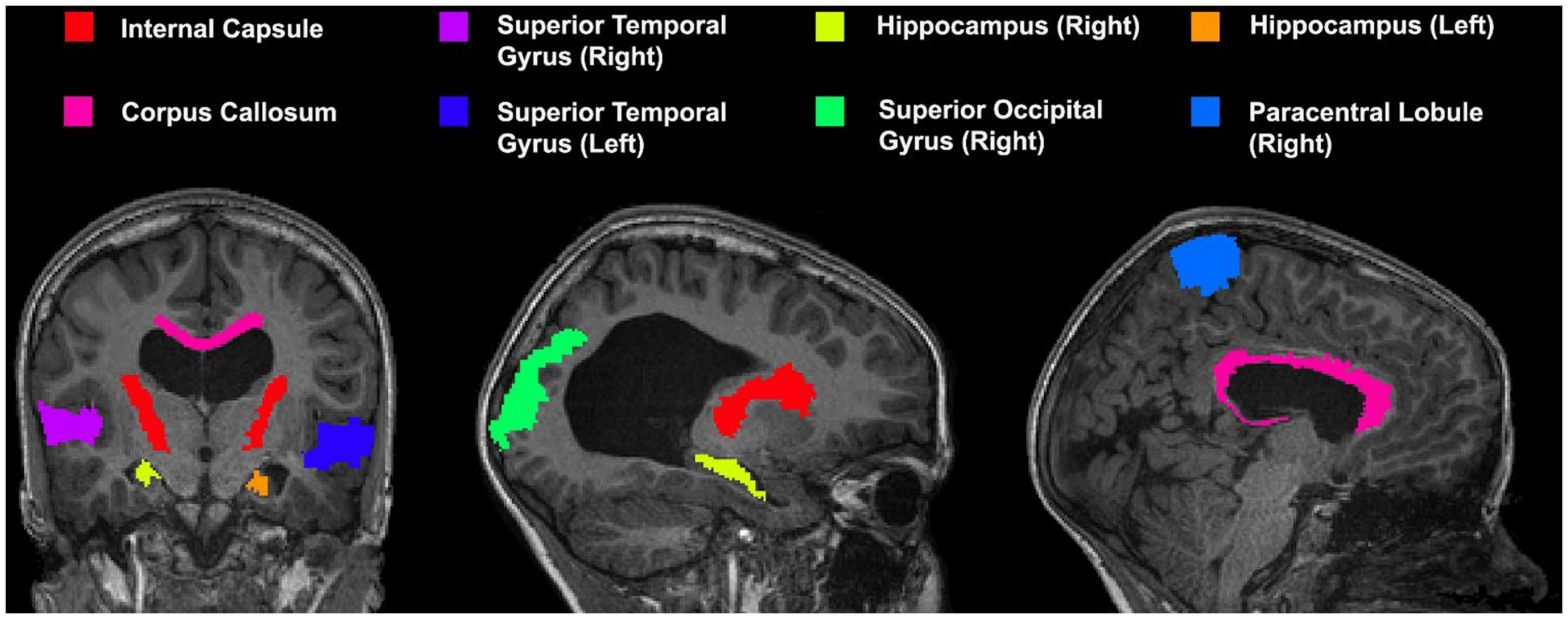
Custom atlas used for registration which includes various cortical and subcortical structures. All selected regions are represented at least once unilaterally.

A series of cortical and subcortical regions were selected to represent areas proximal and distal to the area of deformation as it has been previously demonstrated that registration accuracy can be impacted by proximity to the region of deformation (Ou et al., 2014). Areas included in the custom atlas include the corpus callosum, internal capsule, superior temporal gyrus, hippocampus, superior occipital gyrus, and paracentral lobule (view Figure 3 for the atlas). All areas were manually segmented from each patient’s neuroimage as well as the template image and verified by an expert (SdeR). The custom study atlas in each participant’s native space was then warped using the generated warps from all registrations for analysis.

#### 2.5.2 Computational time

All registrations were performed on a computer with the Linux CentOS version 8 operating system, 64GB of RAM, GeForce 970GTX GPU, and an AMD Ryzen 5 3600 6-Core Processor (3.6GHz/4.2 GHz boost). Registration time was reported in minutes, rounded up to the nearest minute. Given that computation time can be influenced by the size of deformation needing to be estimated, computation time for both the most and least deformed brains have been reported. Multi-core processing was used whenever supported by the software tool and the number of cores used was reported.

#### 2.5.3 Similarity metrics

In order to evaluate the accuracy of the registration two commonly reported similarity metrics were used (Taha and Hanbury, 2015). The warped participant atlas was compared to the same areas segmented from the MNI-152 template image. The Dice coefficients (DICE) were computed for each registration to assess similarity in overlap of the selected 3-dimensional regions (Dice, 1945). Using two sets, A and B, the DICE is defined as:


$$\mathrm{DICE}=\frac{2\;|\left(A\cap B\right)|\;}{\left|A|+|B\right|}$$

Additionally, Hausdorff Distance (HD) which is a measure of spatial distance was also assessed (Hausdorff, 1914). Using two sets, A and B, HD is defined as:


$$HD=\max\left(h\left(A,B\right),h\left(B,A\right)\right)$$

$$h\left(A,B\right)=\max_{a\epsilon A}\min_{b\epsilon B}\left|\left|a-b\right|\right|$$

## 3 Results

### 3.1 Participants

Eight patients with hydrocephalus treated with a VP shunt were included in the current study (1 female, mean age = 8.79 years, sd = 1.81). Their voxel-based ventricle volume ranged from 7,250 mm^3^ to 336,735 mm^3^. The etiology of the hydrocephalus was variable between patients, and included intraventricular hemorrhage, dandy-walker’s malformation, meningitis, and spina bifida. Complete atlas generation was possible in seven of the eight participants. In the participant with the largest ventricle size severe deformities resulted in the inability to distinguish three cortical regions (i.e., left superior occipital gyrus, and both left and right paracentral lobules).

### 3.2 Normalization

A total of 592 registrations were performed. Excluding the registration of whole-brain bias corrected data, registrations directly to the MNI template had a larger DICE and smaller HD, indicative of a more accurate registration, compared to registering to an age-appropriate template prior to the MNI-152 template.

**Figure 4:**
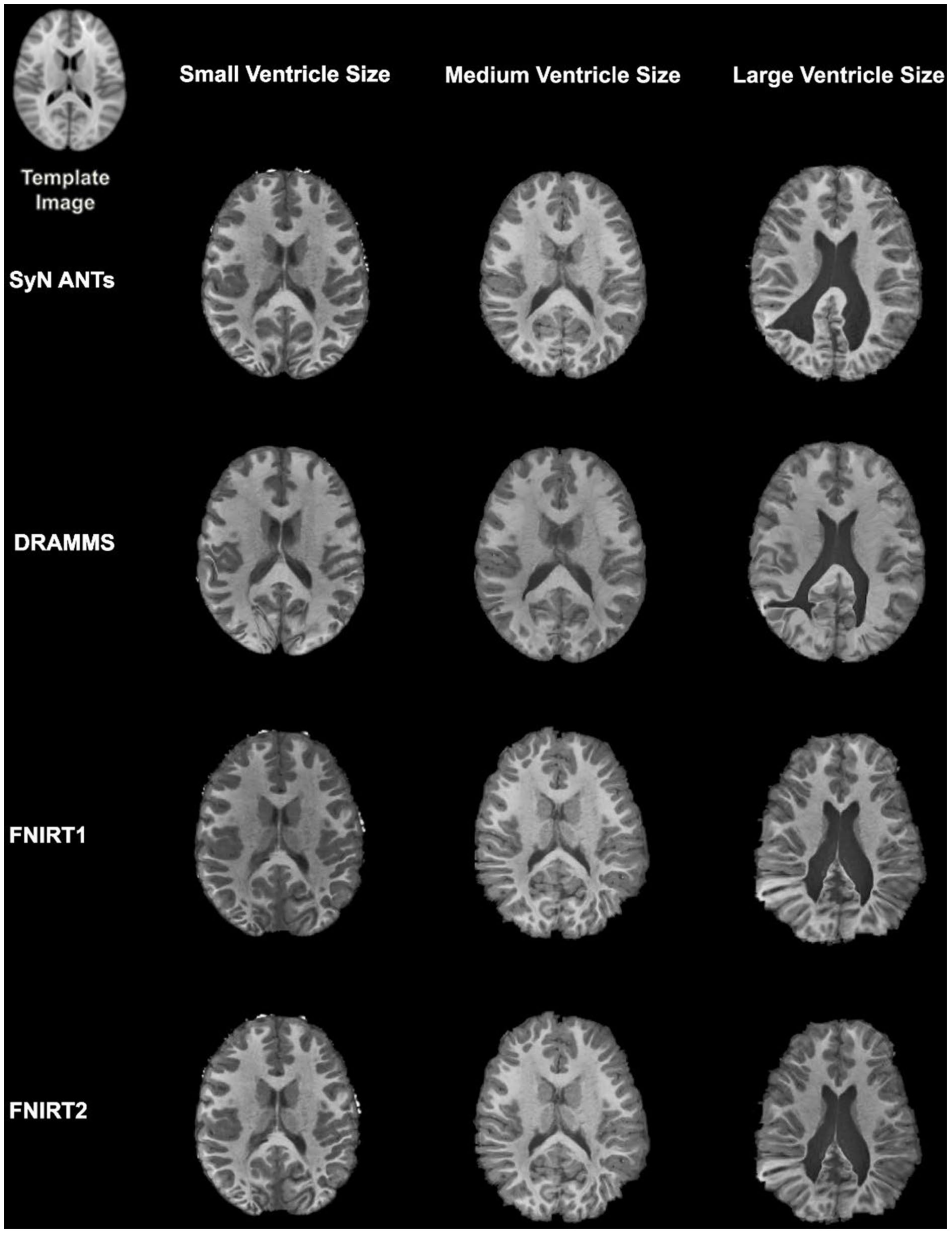
Normalization results for three participants with different ventricle sizes. The participants’ neuroimages were skull-stripped and bias corrected prior to undergoing normalization.

Overall, normalization performed with the preprocessing steps of both skull-striping and bias correction had a larger DICE (*median DICE =* 0.5810, *IQR =* 0.1740) and smaller HD (*median HD =* 12.2915, *IQR =* 5.2510) compared to those normalizations with whole-brain, and non-bias corrected neuroimages. Figure 4 qualitatively shows registrations for skull-stripped bias corrected images.

**Figure 5:**
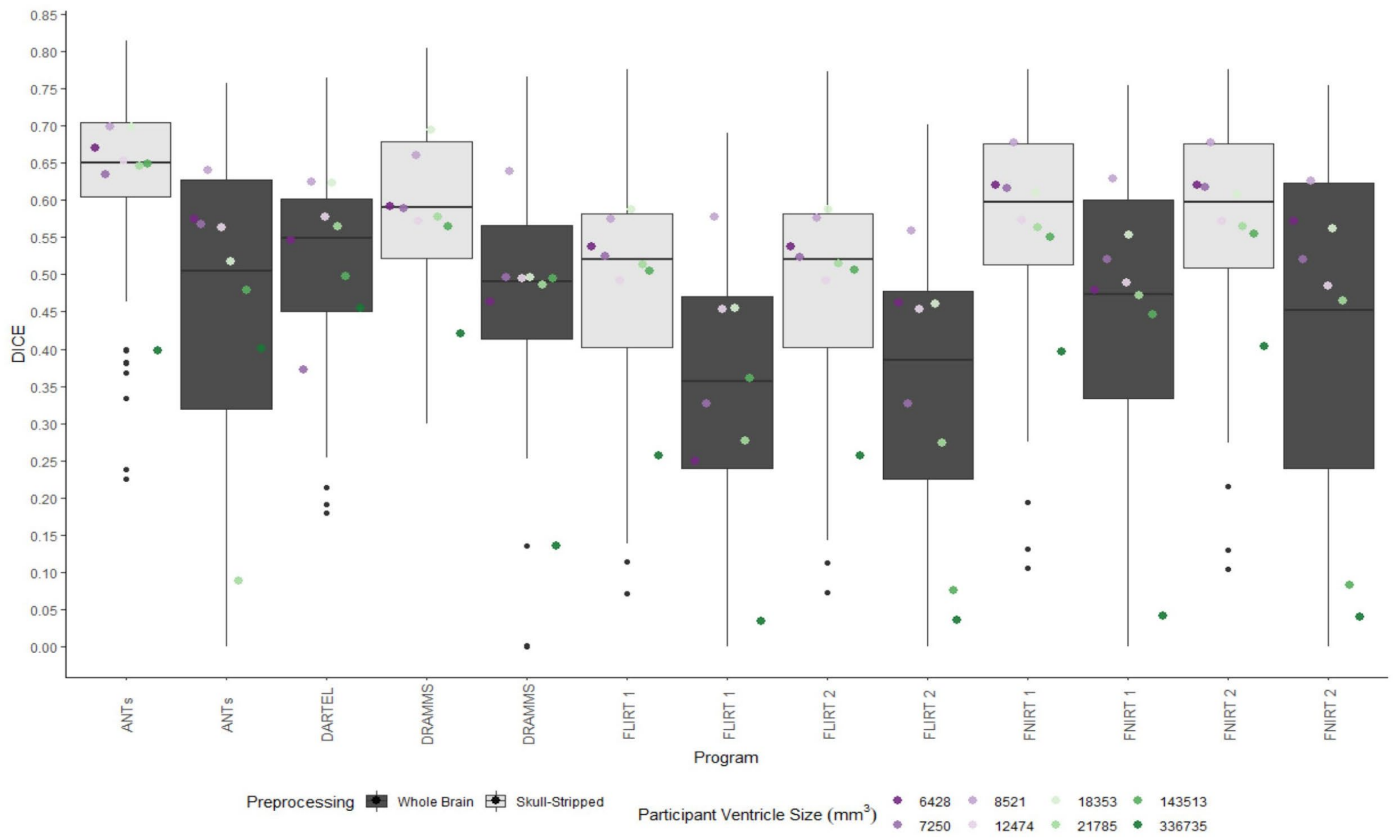
Box plots of the DICE scores per program for normalization from participant T1 to the MNI-152 template image. All images were bias corrected. Results from both whole brain and skull-stripped images are shown in dark gray and light gray, respectively. The median DICE per participant is plotted by ventricle size.

**Figure 6:**
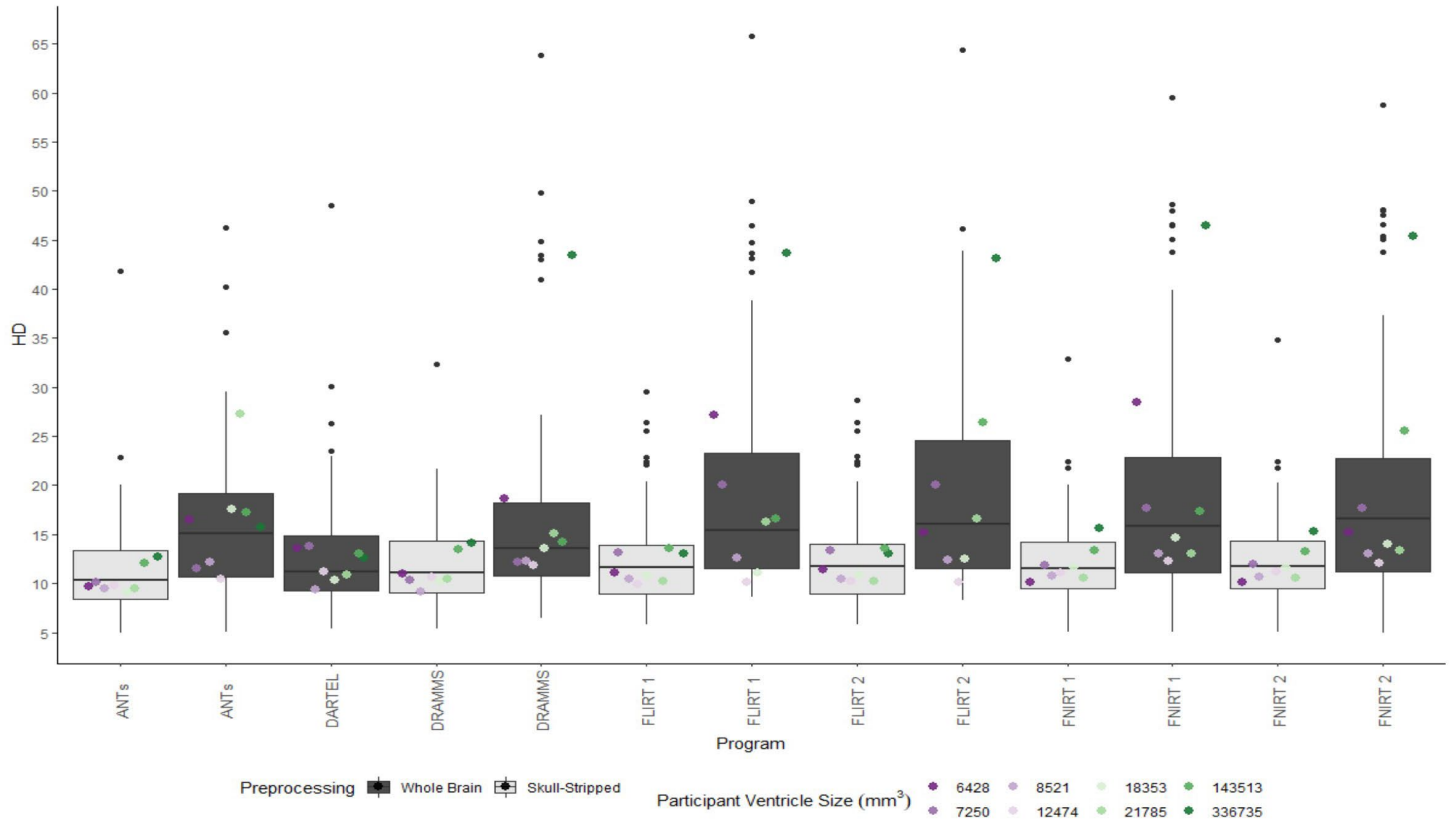
Box plot of the HD per program for normalization from participant T1 to the MNI-152 template image. All images were bias corrected. Results from both whole brain and skull-stripped images are shown in dark gray and light gray, respectively. The median HD per participant is plotted by ventricle size.

**Figure 7:**
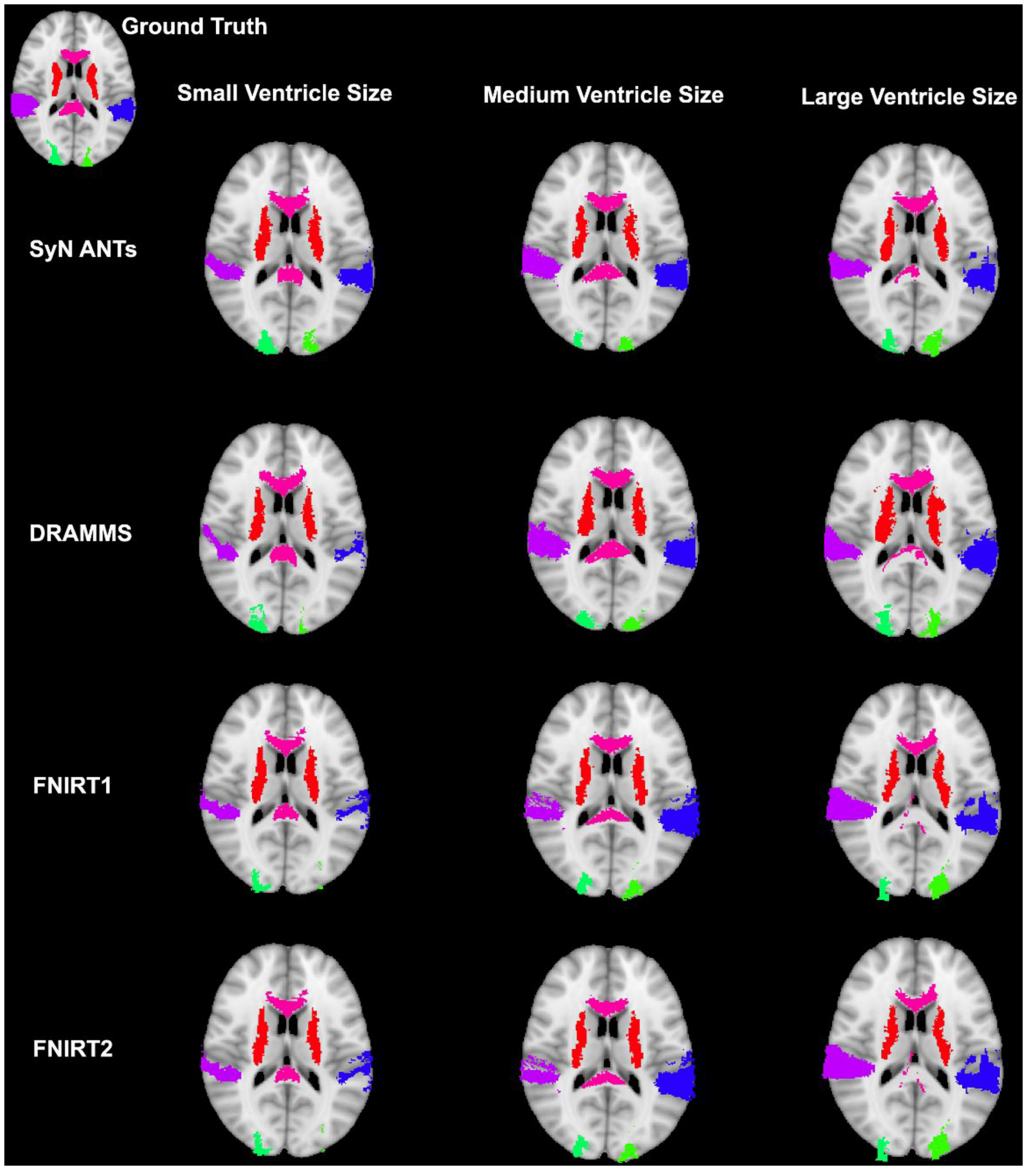
Results from three participants with varying ventricle sizes. The warped atlases for each participant have been overlayed onto the MNI-152 template. All were preprocessed with brain extraction and bias correction and the participant’s image was registered directly to the MNI-152 template.

A similar pattern was observed in those registered first to an age-appropriate template where the largest DICE (*median DICE =* 0.5637 *IQR =* 0.1900) was observed in images which underwent skull-stripping and bias correction. Table 2 outlines the median DICE and HD for all regions in the study atlas across all programs and the various preprocessing steps. Figure 5 depicts box plots for the DICE score per program and Figure 6 depicts box plots for the HD per program. Both figures use the preprocessing step of bias correction and include results with, and without skull-stripping. The median score for each participant has been shown by ventricle size. Qualitative results for three participants (small, medium, and large ventricle size) have been depicted for skull-stripped, bias corrected in Figure 7.

**Table 2:** Summary statistics for normalization accuracy across 50 registration conditions.

Preprocessing	Similarity metric	ANTs	DARTEL	DRAMMS	FLIRT *(1)*	FLIRT *(2)*	FNIRT *(1)*	FNIRT *(2)*	Overall
		Median(IQR)	Median(IQR)	Median(IQR)	Median(IQR)	Median(IQR)	Median(IQR)	Median(IQR)	Median(IQR)
*T1 to MNI152*									
Whole brain	DICE	0.5102(0.2967)	0.5541(0.1604)	0.4979(0.1796)	0.3957(0.1824)	0.4110(0.1763)	0.5008(0.2074)	0.5205(0.2057)	0.4767(0.2321)
	HD	14.3180(8.7530)	11.5330 (5.2630)	14.3530(6.3730)	14.7990(10.0950)	14.7990 (8.4870)	14.3530(8.4113)	14.8660(8.0610)	14.1770(7.7735)
Whole brain bias corrected	DICE	0.5051(0.3078)	0.5388 (0.1624)	0.4906(0.1521)	0.3568 (0.2304)	0.3857(0.2522)	0.4738(0.2675)	0.4518(0.3846)	0.4506(0.2602)
	HD	15.0330(8.5790)	11.4020(4.7974)	13.5650(7.4310)	15.3950(11.7050)	16.0310(13.0030)	15.8430(11.7800)	16.6130(11.4690)	14.4570(10.3255)
Skull-stripped	DICE	0.6468(0.1389)	–	0.5991(0.1577)	0.5291(0.1745)	0.5290(0.1769)	0.5765(0.1981)	0.5778(0.1976)	0.5734(0.1852)
	HD	10.6300(5.6240)	–	11.0450(5.0985)	11.3580(4.9522)	11.3580 (4.8062)	11.7900(5.5866)	11.7900(5.6756)	11.1800(5.5638)
Skull-stripped bias corrected	DICE	0.6504 (0.1009)	–	0.5909(0.1571)	0.5201(0.1796)	0.5211(0.1787)	0.5974(0.1617)	0.5969(0.1674)	0.5810(0.1740)
	HD	10.3920(4.9754)	–	11.0910(5.3180)	11.6620(4.9837)	11.7050(5.0917)	11.5760(4.8140)	11.7900(4.8840)	11.2915(5.2510)
*T1 to NIHPD, NIHPD to MNI152*									
Whole brain	DICE	0.4928(0.2906)	-	0.4485(0.1737)	0.4480(0.2294)	0.4473(0.2425)	0.4987(0.2521)	0.5039(0.2933)	0.4700(0.2380)
	HD	13.4910(9.2300)	–	15.9370(6.8720)	15.2640(6.6720)	15.2970(6.8540)	15.2640(8.7650)	15.1660(8.0260)	15.2640(7.8088)
Whole brain bias corrected	DICE	0.5303(0.2716)	–	0.4563(0.2033)	0.4438(0.2280)	0.4520(0.2266)	0.4969(0.2383)	0.4950(0.2365)	0.4688(0.2352)
	HD	13.8920(9.1050)	–	14.7650(5.1040)	15.0670(7.1490)	15.2640(7.2100)	14.3180(7.9180)	14.1770(8.2680)	14.5260(7.5223)
Skull-stripped	DICE	0.6439(0.1753)	–	0.5678(0.1496)	0.5285(0.1838)	0.5261(0.1856)	0.5586(0.2090)	0.5532(0.2092)	0.5584(0.1880)
	HD	10.4400(6.0464)	–	12.0830(6.2298)	11.7470 (5.4107)	11.7470 (5.4862)	13.0380(6.5325)	12.7670(6.2940)	11.8740(5.8348)
Skull-stripped bias corrected	DICE	0.6590(0.1449)	–	0.5678(0.1496)	0.5201(0.1790)	0.5190(0.1819)	0.5719(0.2018)	0.5727(0.2133)	0.5637(0.1900)
	HD	9.4340(5.7898)	–	12.0830(6.2298)	11.7470(4.6544)	11.7470(4.4014)	12.3690(5.7525)	12.6890(5.5520)	11.8950(5.8424)

Whether assessed with DICE or HD, the interquartile range is often smaller for bias corrected images that underwent skull-stripping compared to whole brain images for the majority of programs assessed. Additionally, regardless of program and preprocessing performed, patients with the largest ventricle size predominately have poorer registration accuracy compared to those with a smaller ventricle size as measured using DICE. In contrast, when accuracy is measured using HD there is less distinction between accuracy based on ventricle size, though participants with the two largest ventricle sizes (i.e., ventricle size >100,000 mm^3^) often have scores worse than the median.

**Figure 8:**
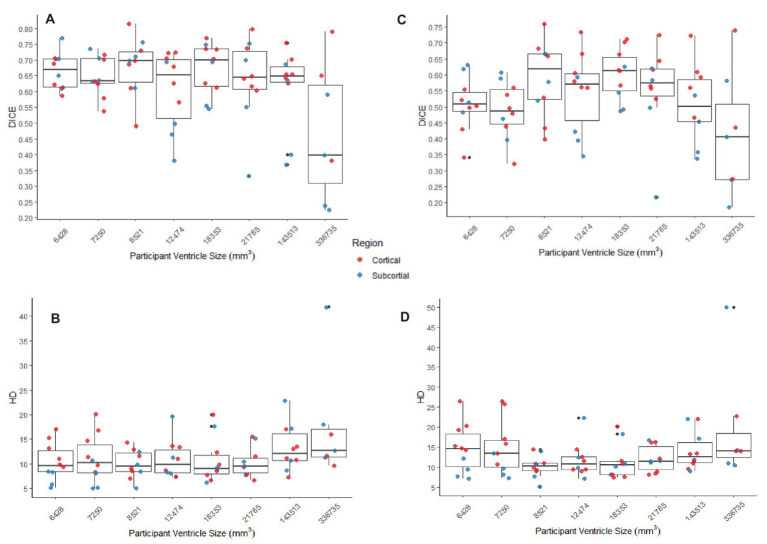
**(A,B)** The DICE and HD, respectively, by participant by region for SyN with the preprocessing steps of skull-stripping and bias correction. **(C,D)** The DICE and HD, respectively, by participant by region for DARTEL with no preprocessing steps (whole-brain, no bias-correction). In all graphs cortical structures are red, and subcortical structures are blue.

**Figure 9:**
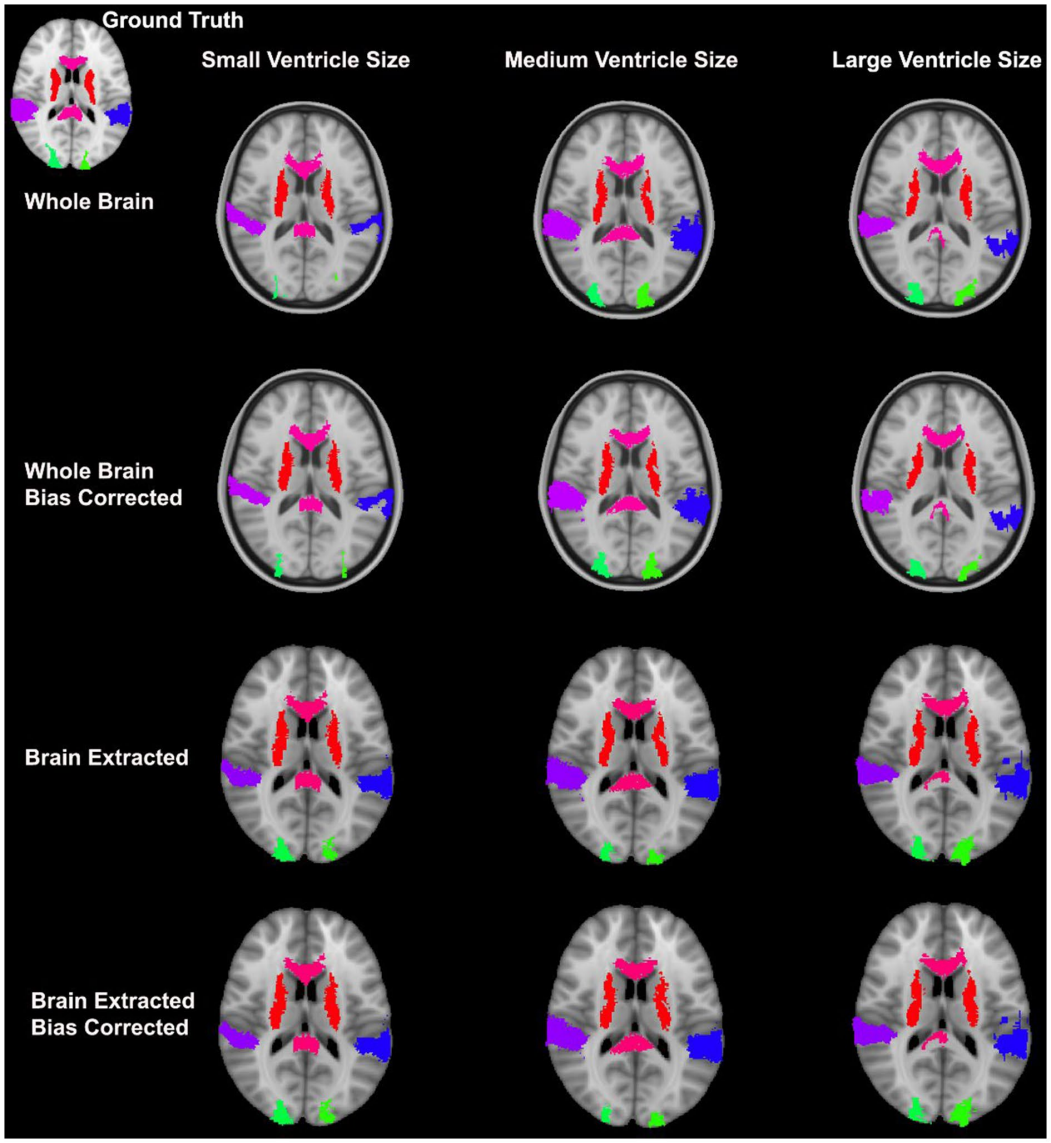
Warped atlases using the SyN algorithm by ANTs. All four preprocessing options with direct registration to the MNI152 template are depicted. Three participants are shown to represent different ventricle sizes. Warps have been overlayed onto the MNI152 template.

The better DICE was seen with the SyN algorithm by ANTs with the preprocessing steps of skull-stripping, and bias correction, with or without initial registration to a pediatric atlas (without intermediate registration *median DICE =* 0.6504, *IQR =* 0.1009; *median HD =* 10.3920, *IQR =* 4.9754; with initial registration to a pediatric atlas *median DICE =* 0.6590, *IQR =* 0.1449; *median HD =* 9.4340, *IQR =* 5.7898). Figures 8A,B shows the individual performance for each participant, and each region of interest using the SyN algorithm with bias correction and skull-stripping, results are depicted qualitatively in Figure 9. As ventricle size increases, overall subcortical regions which are closer to the ventricles, on average, have lower DICE compared to cortical regions. When assessing accuracy using HD, participants with the smallest ventricle sizes (i.e., < 8,000 mm^3^) predominately have a HD for subcortical structures below the median and an HD for cortical structures above the median.

**Figure 10:**
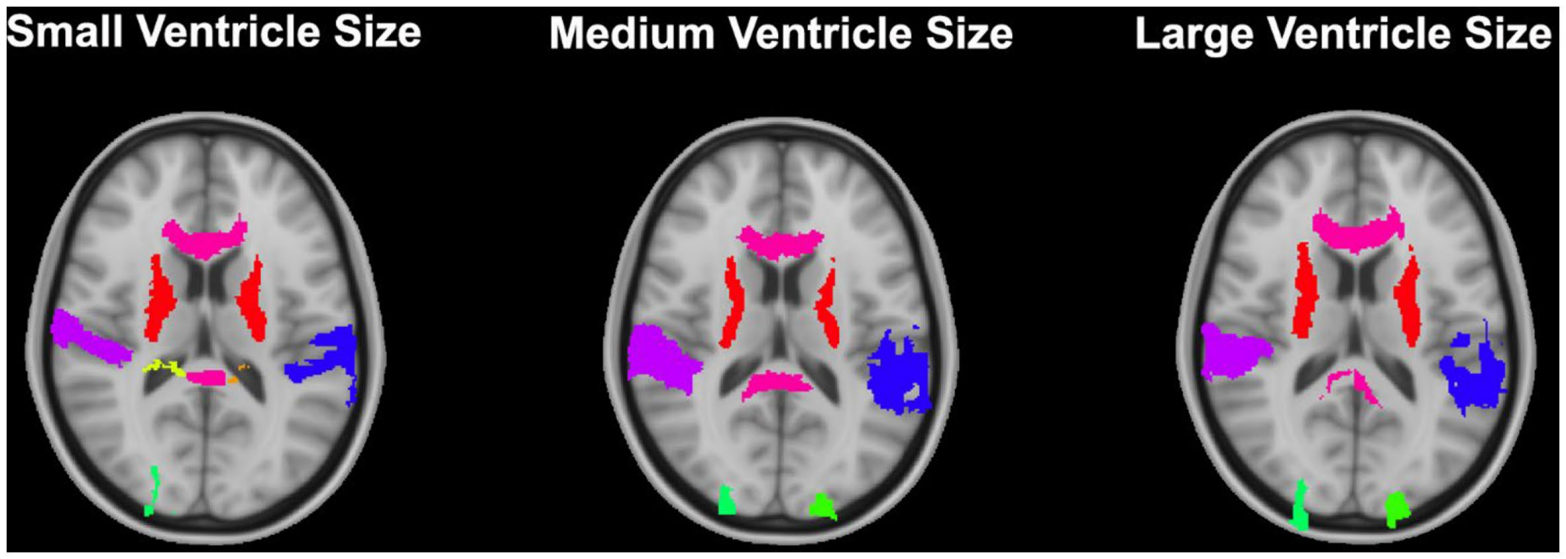
Results from the DARTEL algorithm with no preprocessing (whole brain, no bias correction) for 3 participants with varying ventricle sizes. Results have been overlayed onto the MNI-152 template image.

The best performance with the least number of preprocessing steps (i.e., whole brain, no bias correction) was the DARTEL toolbox by SPM (*median DICE =* 0.5541, *sd =* 0.1604; *median HD =* 11.5330, *sd =* 5.2630). Similar to SyN, subcortical regions generally have a lower DICE (Figures 8C,D) as participant ventricle size increases, compared to cortical regions. Results are depicted qualitatively in Figure 10.

### 3.3 Computational time

Computational time for the smallest and largest ventricle sizes are seen in Table 3. Despite the large difference in volumes, the time to complete the normalization for either participant are similar except for FNIRT wherein the participant with the smaller ventricle size has a much quicker registration to the MNI-152 atlas relative to the participant with the larger ventricle size. The fastest non-linear registration is with ANTs (approximately 18 min). DRAMMS and FNIRT have comparable times (approximately 20 min–40 min) and both use a single-core. Performing two series of registrations from patient T1 to NIHPD, then the registration from the NIHPD atlas to MNI-152 atlas almost increases all the times two-fold which is to be expected as this process involves two-times the registrations. The majority of the algorithms only offer single-core computation. As the DARTEL Toolbox creates a group-wise template, its computational time is dependent on the number of participants. Given the performance of the DARTEL Toolbox with whole-brain non-bias corrected data, this computational time was included.

**Table 3:** Time to completion.

	Smallest ventricle size		Largest ventricle size	
	BET bias corrected (min)	Pediatric atlas BET bias corrected (mins)	BET bias corrected (mins)	Pediatric atlas BET bias corrected (mins)
DRAMMS (single core)	34	83	36	95
FLIRT 1, FSL (single core)	1	2	1	2
FLIRT 2, FSL (single core)	1	2	1	2
FNIRT 1, FSL (single core)	17	64	43	53
FNIRT 2, FSL (single core)	33	63	41	53
SyN, ANTs (single core)	127	273	129	274
SyN, ANTs (10 cores)	18	40	18	39
DARTEL	All participants, whole-brain, no bias-correction:			29

## 4 Discussion

Normalization of neuroimages of pediatric patients with shunt-treated hydrocephalus was assessed using a variety of freely available software tools commonly used for neuroimaging studies. Fifty ways of normalizing neuroimages were examined wherein variations included programs, parameters, and preprocessing steps for a total of 592 registrations performed.

Our study revealed that SyN had the most accurate registration as measured by DICE, and HD with, or without registration to a pediatric atlas. Previous studies assessing the accuracy of registration in healthy brains and/or brains with pathologies have also highlighted SyN as registration algorithms that performs with high accuracy (Klein et al., 2009; Ripolles et al., 2012; Ou et al., 2014). While only few studies have focused on registration in pediatric populations, there has been interest in registration in pathological adult populations. For example, in the study conducted by Ou et al. (2014) databases including patients with Alzheimer’s Disease and brain tumors were assessed. Notably parallels can be drawn between the aforementioned datasets, and the current study’s dataset. In specific, Alzheimer’s Disease patient can present with larger ventricles, and brain tumors are a non-correspondence when compared to healthy brain images. Similar to the current study, SyN by ANTs had high accuracy in these pathological populations. Additionally, DRAMMS was one of their best performing algorithms. In contrast, in the current study, DRAMMS was outperformed by other algorithms including SyN. Similarly in a study which assessed the normalization of deep brain structures in adults who underwent neurosurgery, SyN outperformed all other assessed algorithms (Vogel et al., 2020). Furthermore, in a study assessing surface and volume registration in healthy pediatric brains, ANTs also outperformed other volume registration techniques (Ghosh et al., 2010). The SyN algorithm has been identified as robust when faced with different non-pediatric datasets, and this may be due to its large degree of freedom (Klein et al., 2009; Ou et al., 2014). Further, Ou et al. (2014) suggested that the decrease in registration accuracy observed particularly in the dataset with Alzheimer’s Disease patients could be due to variable degrees of neurodegeneration (e.g., various ventricle sizes, brain structure sizes, and atrophy). Indeed the potential limitations caused by that dataset is similar to the problem-set in the current study.

In the current study, we found superior registration accuracy using bias corrected, skull-stripped data; however, normalization with non-preprocessed data (i.e., whole-brain, non-bias corrected) can also result in normalized images with good accuracy. Typically, performing bias correction can help to improve normalization accuracy and it is non-computationally intensive relative to the time required to perform registrations. In addition to having a small benefit for normalization, bias correction has been shown to improve brain extraction (Fennema-Notestine et al., 2006). The current dataset composed of pathological pediatric neuroimages revealed that the removal of the skull has been shown to be incredibly beneficial for normalization. However, it is worth noting that skull-stripping has been identified as a non-trivial task which could be very time consuming (Popescu et al., 2012). Poor brain extractions can result in removing areas of the brain or including non-brain matter. Furthermore the presence of neck in the volumes have been shown to negatively impact brain extraction (Popescu et al., 2012). These errors in brain extraction could result in poor registration wherein the non-brain matter for example, could be interpreted as brain matter.

Towards minimizing preprocessing steps, whole brain normalization can also be performed in patients with complex neuropathology, such as that seen in hydrocephalus. It was demonstrated that the DARTEL toolbox outperforms many of the other algorithms under these circumstances. The DARTEL toolbox makes use of groupwise registration and is the only tool assessed in this study which uses this process (Ashburner, 2007). In this case, a group-specific template is created based on the whole input dataset, then each participant’s neuroimage is then registered to the group template. Group-wise registrations are beneficial as there is no *a priori* template selection required; however, performing group-wise registration between different groups (e.g., healthy controls compared to patients with morphological differences) is non-trivial and an area of interest (Liao et al., 2012; Ribbens et al., 2013).

Given the differences between pediatric and adult neuroimages such as the size, shape, and tissue type, it has been previously suggested that using an age-appropriate brain template in registrations, can help to improve registrations reducing the age-based variability between images (Courchesne et al., 2000; Fonov et al., 2011). We have demonstrated that with our current dataset, that registering to an age-appropriate template, for the most part, did not improve registration. Accuracy was similar whether an age-specific template was used, though accuracy was slightly reduced overall. Given the purpose of an age-specific template is to better represent a pediatric brain, structural changes to the brain due to hydrocephalus may make these registrations more difficult (Del Bigio, 2010). In addition, there was almost a two-fold increase in processing time with age-based registration compared to a single registration between the participant’s image and the target MNI-152 image, which may not be ideal in some circumstances. Therefore, while we would still advise to register healthy participants with age-specific templates, this step can be skipped when registering children with large anatomical deformation due for example to hydrocephalus.

Regardless of the overall accuracy of the registration (measured using DICE), often, participants with larger ventricle sizes had poorer normalization accuracy compared to those with smaller ventricle sizes. Further, the areas that were most impacted as measured by DICE were those near the ventricles (i.e., subcortical regions) such that these areas have low overlap with ground truth. This observation may be due to the sensitivity of the DICE when comparing regions of different sizes, wherein the size of subcortical structures, particularly the ones chosen, are much smaller in volume compared to the cortical structures. The inclusion of subcortical brain structures in neuroimages studies can be challenging given their small size and many are already excluded from standard atlases (Forstmann et al., 2016). To address this, some studies have used a modified DICE, specifically dilated DICE when assessing sub-cortical structures (Bazin et al., 2020).

Notably, many of the assessed programs do not make use of multicore computing for a single subject. Only SyN allowed a streamline method of utilizing multiple cores by modifying the function call^4^ (Avants et al., 2009). Improving the computational efficacy of registrations is an area of interest. Given their time-consuming nature, registrations are often performed outside of busy clinical practice, though they have a utility in clinical/surgical practice (Alam et al., 2018). Ultimately, there is ongoing interest in utilizing the power of modern GPUs which are built for parallel processing to improve the computational efficiency of image registration (Shams et al., 2010).

Normalizing images of pediatric patients with shunt-treated hydrocephalus provide a unique opportunity to assess the accuracy of various non-linear registration programs given many different challenges. While the patients in this study had a wide range of ventricle sizes, our study was limited by sample size. Having a larger sample size would potentially allow us to better understand the impact of large deformities on normalization outcomes. Furthermore, as we used a custom atlas for assessing registration accuracy, many regions were excluded. Given a more robust atlas, we could have further assessed the impact of the shunt location on registration accuracy in nearby areas as registration performance can vary based on proximity to a pathological site (Ou et al., 2014). Given the largest DICE being marginally over 0.60, more robust registration algorithms are needed to better account for complex pathologies. Notwithstanding, it is worth noting that all the programs we have used have parameters that can be tuned, and that there are various techniques such as a masking that could have been used to better accommodate our data; however these processes were outside the scope of the current study. Finally, though our best overlap value was not very large, this is consistent with various results from other studies who have assessed potential challenging registrations (Ghosh et al., 2010; Ou et al., 2014; Vogel et al., 2020). With the performance of these programs in complex datasets, it is therefore important to complete visual checks following registration and consider manual segmentation of areas of interest when possible.

In sum, we assessed four different non-linear registration algorithms to normalize neuroimages from pediatric patients with shunt-treated hydrocephalus. Ultimately preprocessing the neuroimages to remove non-brain tissue (e.g., skull-striping) and bias correcting resulted in on average the most accurate normalized images using the SyN algorithm. Notably, various other studies have demonstrated good registration accuracy with SyN in non-pediatric populations, which suggest that SyN is a robust normalization algorithm under a variety of circumstances (Klein et al., 2009; Ghosh et al., 2010; Ou et al., 2014). We also demonstrated that the DARTEL Toolbox, which performs a group-wise registration, can produce a similarly accurate registration without any preprocessing steps. Finally, while registering to an age-appropriate atlas has been shown to produce a superior registration outcome, overall it did not have a positive impact on the registration accuracy in the current study. These results may help to inform a normalization pipeline and algorithm selection for studies with pediatric patients and complex neuronal pathologies.

## Data availability statement

The datasets presented in this article are not readily available because we do not have a data sharing agreement with our participants. Requests to access the datasets should be directed to the corresponding author, sderibau@uwo.ca.

## Ethics statement

The studies involving humans were approved by Western research ethics board—health sciences REB. The studies were conducted in accordance with the local legislation and institutional requirements. Written informed consent for participation in this study was provided by the participants’ legal guardians/next of kin.

## Author contributions

R-MR: Conceptualization, Formal analysis, Investigation, Methodology, Visualization, Writing – original draft, Writing – review & editing. RE: Conceptualization, Methodology, Supervision, Writing – review & editing. SR: Conceptualization, Methodology, Supervision, Writing – review & editing.

## Footnotes

^1^ https://fsl.fmrib.ox.ac.uk/fsl

^2^ https://www.fil.ion.ucl.ac.uk/spm

^3^ http://picsl.upenn.edu/software/ants/

^4^ http://stnava.github.io/ANTs/
