# Supplementary material for: Evaluating normalized registration and preprocessing methodologies for the analysis of brain MRI in pediatric patients with shunt-treated hydrocephalus
Source: Front Neurosci. 2024 May 30;18:1405363. doi: 10.3389/fnins.2024.1405363 (PMC11182356; doi:10.3389/fnins.2024.1405363)
Supplement: Supplementary file 1 [file Data_Sheet_1.docx]

Appendix 1

SyN ANTs:

-d 3

-f templateImage

-m participantImage

-o conditionLabel

-t s

-r 4

-s 26

-g 0.1

-p d

-j 0

-y 0

-z 1

DARTEL Toolbox:

Segmentation Step:

Data

Volumes: participantImages

Bias regularisation: no regularisation

Bias FWHM: No correction

Save Bias Corrected: Save Nothing

Tissue

Tissue probability map: TPM.nii, 1

Num. Gaussians: 1

Native Tissue: Native + Dartel Imported

Warped Tissue: None

Tissue probability map: TPM.nii, 2

Num. Gaussians: 1

Native Tissue: Native + Dartel Imported

Warped Tissue: None

Tissue probability map: TPM.nii, 3

Num. Gaussians: 2

Native Tissue: Native + Dartel Imported

Warped Tissue: None

Tissue probability map: TPM.nii, 4

Num. Gaussians: 3

Native Tissue: Native + Dartel Imported

Warped Tissue: None

Tissue probability map: TPM.nii, 5

Num. Gaussians: 4

Native Tissue: Native + Dartel Imported

Warped Tissue: None

Tissue probability map: TPM.nii, 6

Num. Gaussians: 2

Native Tissue: Native + Dartel Imported

Warped Tissue: None

Warping & MRF

MRF Parameter: 1

Clean Up: Light Clean

Warping Regularisation: 1x5 double

Affine Regularisation: ICBM space template – European brains

Smoothness: 0

Sampling distance: 3

Deformation Fields: None

Run Dartel (create Template)

Images: participantImages

Settings

Template basename: Template

Regularisation Form: Linear Elastic Energy

Outer iterations

Inner Iterations: 3

Reg params: [4 2 1e-06]

Time Steps: 1

Smoothing Parameter: 16

Inner Iterations: 3

Reg params: [2 1 1e-06]

Time Steps: 1

Smoothing Parameter: 8

Inner Iterations: 3

Reg params: [1 0.5 1e-06]

Time Steps: 2

Smoothing Parameter: 4

Inner Iterations: 3

Reg params: [0.5 0.25 1e-06]

Time Steps: 4

Smoothing Parameter: 2

Inner Iterations: 3

Reg params: [0.25 0.125 1e-06]

Time Steps: 16

Smoothing Parameter: 1

Inner Iterations: 3

Reg params: [0.25 0.125 1e-06]

Time Steps: 64

Smoothing Parameter: 0.5

Optimisation Settings

LM Regularisation: 0.01

Cycles: 3

Iterations: 3

Normalize to MNI Space

Dartel Template: templateImage

Voxel sizes: [1 1 1]

Bounding box: 2x3 double

Preserve: Preserve Amount

Gaussian FWHM: [8 8 8]

DRAAMS:

-S participantImage

-T templateImage

-O outputRegisteredImage

-D outputDeformationField

FLIRT

-in participantImage

-ref templateImage

-out outputImage

-omat outputMatrix

-cost corratio

-searchcost corratio

-anglerep euler

-interp trilinear

-sincwidth 7

-bins 256

-dof 12

-searchrx -90 90 FLIRT2 uses -10 10

-searchry -90 90 FLIRT2 uses -10 10

-searchrz -90 90 FLIRT2 uses -10 10

-coarsesearch 60

-finesearch 18

FNIRT

-ref templateImage

-in participantImage

-aff affineTransformation

-iout outputImage

-fout outputField

-cout outputFiledCoefficients

-applyrefmask 1

-applyinmask 1

-imprefm 1

-impinm 1

-imprefval 0

-impinval 0

-miter 5,5,5,5

-subsamp 4,2,1,1

-warpres 10,10,10

-splineorder 3

-infwhm 6,4,2,2

-reffwhm 4,2,0,0

-regmod bending_energy

-ssqlambda 1

-jacrange 0.01, 100.0

-refderiv 0

-intorder 5

-biasres 50,50,50

-biaslambda 10000

-estint 1

-numprec double

-interp linear
